# The Association of Epstein-Barr Virus With Cancer

**DOI:** 10.7759/cureus.26314

**Published:** 2022-06-25

**Authors:** Pragnesh D Patel, Rahmah Alghareeb, Afshan Hussain, Marvi V Maheshwari, Nabeeha Khalid

**Affiliations:** 1 Research, Saint George's University, School of Medicine, St. George's, GRD; 2 Research, College of Medicine, University of Baghdad, Baghdad, IRQ; 3 Research, Dow Medical College and Dr. Ruth K. M. (Katherina Martha) Pfau Civil Hospital, Karachi, PAK; 4 Research, Our Lady of Fatima University College of Medicine, Valenzuela, PHL; 5 Cardiology, Omar Hospital and Cardiac Center, Lahore, PAK

**Keywords:** ebv, nasopharyngeal carcinoma, burkitt's lymphoma, hodgkins lymphoma, epstein-barr virus

## Abstract

Epstein-Barr virus (EBV) is classified as a herpesvirus and is known for being one of the few viruses that can lead to the development of cancer. This study has gathered several studies to provide evidence as to this association as well as some of the mechanisms specific to EBV that allow this to happen. The development of EBV into cancer as well as the proteins involved in this oncogenesis play a crucial role in understanding this problem as well as creating a solution for mitigating this disease process in the future. This study summarized three of the most common malignancies caused by EBV in order to consolidate information about each of them. Additional emphasis was placed on finding which EBV serum markers were seen to be most indicative of prognosis and likelihood of developing malignancy. Higher serum EBV viral DNA loads were seen to be a useful indicator in assessing the risk of various cancers and should be studied further in relation to cancers that were not mentioned in this review.

## Introduction and background

Epstein-Barr virus (EBV) was first discovered somewhat incidentally in the 1960s when a researcher studying Burkitt's lymphoma was able to culture lymphoma cells in vitro for the first time. Subsequent examination with the then-controversial electron microscope showed the viral particles we now know as EBV [1]. EBV maintains a massive prevalence, with most sources saying that it infects over 90% of the world’s population [2]. EBV has been classified as part of the herpesvirus family, which is signified by its DNA core with an icosahedral capsid [2]. Additionally, humans are the only known host for EBV, which is transmitted from host to host via salivary contact [2]. In adolescents, EBV is the most common cause of infectious mononucleosis, which classically presents with fatigue, sore throat, splenomegaly, and cervical lymphadenopathy [3-5]. Following primary infection, EBV has the ability to cause the production of memory B cells which can harbor the virus in a latent manner [6]. Because of this, patients infected with EBV will be at risk of reactivation of this latent infection in times of stress, infection, or immunosuppression. Though reactivation of the latent virus is cause for concern, the most daunting long-term sequelae of EBV are malignancies such as nasopharyngeal carcinoma and Hodgkin’s lymphoma. Once EBV establishes latency, it becomes unique in comparison to other viruses because of the genes that it encodes. EBV gene variants such as EBNA and latent membrane protein 2a (LMP-2A) have been found to be the reason behind the differentiation of primary B cells to lymphoblastoid cell lineages [7]. For example, the EBNA gene (which has several alternate forms) primarily functions to allow for efficient transcription, while also ensuring persistence of the viral genome in replicating cells [7]. LMP-2A is highly associated with development of lymphoma as it encodes for activation of breakpoint cluster signaling (BCR) independent of antigen molecules, which will then serve to transactivate human endogenous retrovirus (HERV-K18) which functions to create a massive T-cell response [8]. The aim of this review article was to underline the association between EBV and various types of cancer as well as detail which specific proteins are involved in the pathogenesis.

## Review

Method

An English language limited search was conducted on PubMed using the terms "EBV and nasopharyngeal cancer," "EBV and Burkitt's lymphoma," as well as "EBV's association with Hodgkin's lymphoma," in order to find applicable information. Selected studies were limited to those which were published between 1997 and the present so as to sample more recent data with the most up-to-date information. The studies which were used included a variety of reviews, original research, and meta-analyses. The authors reviewed these various types of literature in order to ascertain the connection between EBV and cancer.

Discussion

Epstein-Barr Virus and Nasopharyngeal Carcinoma

Nasopharyngeal carcinoma (NPC) is a malignancy most commonly found in South Asia, the Middle East, and North Africa. It is known for having a variety of risk factors foremost of which are genetic and environmental [9]. Genetic risk factors of NPC involve an affected first-degree relative (which quadruples risk) as well as the inheritance of certain human leukocyte antigen (HLA) genes [9]. Diet and smoking habits can be thought of as important examples of environmental risk factors for NPC; however, this discussion will mainly focus on the contributions of EBV to this pathology [9]. During primary infection, EBV is known to infect the epithelial cells of the nasopharynx as well as circulating B cells depending on the type of surface glycoproteins (gp) the virus is expressing [10]. NPC is characterized by the neoplastic modification of epithelial cells so it is of paramount importance to understand how these cell lines are affected by EBV. One way that EBV infection leads to NPC is by epigenetic modification of the host genome in such a way as to promote unregulated tumor growth [11]. This is primarily accomplished by histone modification and DNA hypermethylation [11,12]. These alterations serve various purposes in promotion of tumor growth, such as downregulating tumor suppressor genes, reducing normal protein transcription, and inactivating DNA repair enzymes [11,12]. EBV is also known to cause genomic instability which can occur when EBV switches between its lytic and latent phases. These phases are heavily influenced by the BZLF1 gene which arrests cell cycle development, and the BRLF1 gene which can cause the cell to inappropriately reenter the S phase causing problems in chromosomal segregation [13,14]. EBV, like many cancer-causing agents, has in-built mechanisms to evade the immune system which stymies the physiological response to anti-tumorigenesis. Epstein-Barr encoded small RNAs (EBER) can inactivate genes that are stimulated by interferon as part of the normal immune response [11]. EBV can also encode the EBNA1 antigen which allows for the chemoattraction of regulatory T cells that function to downregulate the immune response to neoplastic NPC cells [15]. These are some of the major mechanisms that researchers have to use to quantify an association between EBV and NPC. A prospective population-based study published in 2011 used a sample of 18,986 subjects from the Guangdong province of China to establish an association between IgA antibodies to VCA and the development of NPC. This study took a baseline measurement of these antibodies and followed up with the subjects 20 years later using health registers to find out about the development of cancer. The results of this study concluded that 125 individuals ended up developing NPC and that ascending EBV titer counts were associated with increased risk of future malignancy [16]. The above study differs from a more recent work published in 2021 which screened 1363 subjects who were seropositive for EBV VCA and EBNA1-IgA. These patients were followed for a seven-year span and the development of NPC was tracked through a local cancer registry. This study found that 30 of its participants were diagnosed with NPC and that risk of malignancy was positively correlated with increased plasma EBV DNA load [17]. While the two studies mentioned above dealt with diagnosis, a 2003 study added prognosis into the mix. This study used 206 Thai participants belonging to several groups. Seventy-nine were diagnosed with NPC and 127 were age-matched controls. The control group was then divided into three subgroups consisting of 47 healthy subjects, 32 subjects with other diseases, and 48 cases of other cancer. These researchers accepted that IgA and IgG antibodies were useful in diagnosing NPC and were trying to see if serum levels had any correlation with prognosis. They were able to measure the levels of these antibodies across the different groups using indirect immunofluorescence assay. They found that increased amounts of serum IgG antibodies to early antigen (EA), IgA antibodies to EA, and IgA antibodies to VCA were all associated with a worse prognosis of NPC [18]. Finally, a 2004 Chinese study sought to determine whether EBV antibodies to IgA/VCA and IgA/EA were correlated with the level of EBV DNA in the plasma. These researchers were using information from previous studies that affirmed plasma EBV DNA was an indicator of NPC staging and prognosis. Real-time polymerase chain reaction combined with enzyme-linked immunoadsorbent assay was used to determine the level of IgA/VCA in 370 individuals that were grouped as follows: 120 with primary NPC, eight with locally recurrent NPC, 21 with metastatic NPC, 76 with radiotherapy-resistant NPC, 60 with NPC in remission, 38 with non-NPC tumors, and a control group of 47 subjects. This study found higher levels of EBV DNA in all of the cancer groups and showed that the levels declined to near zero when the cancer was in remission. However, the EBV IgA/VCA titers were at a high level in all of the NPC groups which led to the conclusion that the plasma EBV DNA levels were more sensitive and specific in terms of monitoring the development of NPC when being compared to the antibody levels [19]. The aforementioned studies relating EBV to NPC have been listed in Table 1.

**Table 1:** Summary of studies linking EBV diagnosis with NPC development. EBV: Epstein-Barr virus; NPC: nasopharyngeal carcinoma; IgG/EA: immunoglobulin G antibody to early antigen; IgA/VCA: immunoglobulin A antibody to viral capsid antigen

Reference	Design	Population	Cases of NPC	Diagnostic Criteria	Conclusion
Chen et al. (2021) [17]	Observational study	1,363 seropositive subjects from Southern China	30	Endoscopies and biopsies	Plasma EBV DNA can be used to ascertain NPC risk within 3 years
Cao et al. (2011) [16]	Prospective population-based	18,986 subjects from Guangdong Province, China	125	Cancer registries as reported by general practitioners	High EBV titers associated with high malignancy risk
Shao et al. (2004) [19]	Comparative study	370 Chinese participants	285	Histologically confirmed at Cancer Center, Sun Yat-Sen University	EBV DNA is better for monitoring NPC than antibody titers
Tiwawech et al. (2003) [18]	-	206 Thai subjects	79	Previously confirmed diagnosis	IgG/EA, IgA/VCA, IgA/EA can be used for diagnosis and prognosis of NPC

Epstein-Barr Virus and Burkitt's Lymphoma

As mentioned at the beginning of this study, the discovery of EBV occurred while a researcher was studying Burkitt's lymphoma, thus establishing a connection at the very time of its discovery. EBV is known to be latently housed in memory B cells after initial infection; however, the mechanism in which this is accomplished is currently under debate [6,20]. One school thought on how this comes to be is known as the germinal center model. This postulates that the cells that are infected by EBV pass through germinal centers, which are the site of B cell proliferation, class switching, and somatic hypermutation [20,21]. Once in the germinal center, the virally transformed cell exhibits the latency 2 phase where EBNA1, LMP1, and LMP2 are all activated to promote the survival of viral cells in the germinal center and facilitate their expulsion as memory B cells [20,21]. The resulting EBV-infected memory B cells are able to inactivate latency genes to exist in a latency 0 phase which will serve to avoid the immune system [20,21]. A second theory on how EBV is able to infect memory B cells is known as the direct infection model. This model was created because it was seen that EBER-positive cells which were extracted from germinal centers were found to have little to no diversity, meaning somatic hypermutation was not occurring normally. This could potentially be explained by EBV having the ability to directly infect memory B cells and causing them to proliferate without taking part in the germinal center reactions which result in somatic hypermutation [21]. The endemic form of BL, which is usually seen in sub-Saharan Africa, is most commonly correlated with EBV, though the sporadic and HIV-related forms may or may not have associations [22,23]. Burkitt's lymphoma (BL) is characterized by a translocation between chromosomes 8 and 14 resulting in an overexpression of the MYC oncogene [24]. There is an increasing amount of evidence that this overexpression may lead to genomic instability as a result of formation of reactive oxygen species (ROS) which will eventually cause double-stranded DNA breaks [25]. Research provided by the Masucci group found that EBV may contribute to the pathogenesis of BL in a similar way as the aforementioned translocation [26]. This group found that the EBNA1 protein was implicated as having a role in inducing DNA damage through formation of ROS [26]. In addition to this translocation, BL exhibits ways to inhibit cellular apoptosis through point mutations, deletion, and methylation [27]. EBV contributes to the survival of these cancer cells by directly inhibiting apoptosis through EBNA1 granting them extra time to acquire additional mutations allowing for further progression of the endemic form of BL [27,28]. A case-control study published in 2019 used a sample population of 300 Ghanaian children, divided equally into case and control groups, to see which specific EBV antibodies were most often seen in cases of BL. They used a protein microarray as well as statistical analysis to measure levels of antibodies to over 200 sequences of the EBV proteome in order to see if there were any significant markers that could be associated with BL. They found significant increases in 33 different IgG antibodies, with the most pronounced elevations being in BMRF1, BZLF1, and BBLF1 [29]. While the previous study highlighted which antibodies present in the blood may be the most predictive of malignancy, the next study described explores a possible synergistic relationship between malaria and EBV in the pathogenesis of BL. A 2007 Ugandan case-control study separated 325 children under the age of 15 years who had been diagnosed with BL as their case group and used 579 healthy children of the same age as their control group. Serological analysis of the case group was performed in addition to surveying the parents of the participants and from this data, an odds ratio was calculated. This study found that participants with high antibodies against EBV as well as high titers of malaria were five times more likely to develop BL [30]. Additionally, this conclusion could serve to direct future initiatives on prioritizing widespread measures in malaria prevention and treatment due to this synergistic association. A cross-sectional study published in 2019 wanted to see which EBV antibodies were most likely to be seen in patients with histologically confirmed BL. Serum was tested for antibodies using enzyme-linked immunosorbent assay (ELISA), and it was seen that 93% of the participants were positive for EBV IgG antibodies while 86.7% were positive for EBER1 antibodies. Additionally, it was seen that 100% of those who developed jaw tumors (due to the endemic form of BL) were positive for EBER1 antibodies and that those who had this antibody were 1.4 times more likely to develop malignancy [31]. The small percentage of cases that were negative for both of the mentioned EBV antibodies could suggest an alternate mechanism of pathogenesis and could be further researched in the future. A 2020 study involved 58 BL cases and 40 healthy Kenyan children as the control group. These researchers wanted to further the understanding of EBV’s association with BL by determining which variant of EBV (type 1 vs. type 2) was more likely to cause tumorigenesis. They found that the EBNA2-carrying EBV type I was much more prevalent in the experimental group, which even further specifies the link between EBV and BL [32]. The studies associating EBV and BL are condensed in Table 2.

**Table 2:** Summary of included studies linking Epstein-Barr virus and Burkitt's lymphoma. EBV: Epstein-Barr virus; BL: Burkitt's lymphoma; anti-EBV IgG antibodies: immunoglobulin G antibody to EBV; EBER-1: Epstein-Barr virus encoded small RNAs

Reference	Design	Population	Cases of BL	Diagnostic criteria	Conclusion
Kaymaz et al. (2020) [32]	Case-control	98 children 2-14 years of age	58 confirmed cases	-	EBV type 1 was more common subtype in BL cases
Coghill et al. (2020) [29]	Case-control	300 children 0-17 years old	150 confirmed cases	Histological/cytological analysis	33 cases were seen to have elevated anti-EBV IgG antibodies
Ndede et al. (2019) [31]	Cross-sectional	33 children <18 years old	33 confirmed cases	Clinically and histologically confirmed	All BL jaw tumors and 86.7% of other BL tumors carried EBER-1 antigen
Carpenter et al. (2008) [30]	Case-control	904 children <15 years old	325 confirmed cases	Histological analysis	EBV may exhibit synergism with malaria in causing BL

EBV and Hodgkin’s Lymphoma

Hodgkin’s lymphoma (HL) is a rare malignancy of B cells that has an annual incidence of over 9,000 patients [33]. Cellularly characterized by the “owl-eyed,” Reed-Sternberg (RS) cells and consisting of several subtypes, HL is yet another malignancy that can trace aspects of its pathogenesis back to EBV [33,34]. RS cells are multinucleated cells that originate from B cells; however, they have lost most of the observable characteristics normal B cells display [35]. Additionally, they are known to have unregulated activation of proinflammatory cascades that have been postulated to contribute to the pathogenesis of HL [35,36]. In EBV-positive HL, there are specific anti-apoptotic mutations that are not otherwise present in EBV-negative HL [37]. Secondly, EBV is able to rescue BCR-negative cells that are scheduled to undergo apoptosis, and because of this some of these cells are able to become RS cells [37,38]. EBV’s latent genes also contribute to the pathogenesis of HL. EBNA1 is able to suppress transforming growth factor-beta (TGF-β) target protein tyrosine phosphatase receptor kappa whose normal function is to prevent the growth and development of RS cells [39,40]. LMP1 is another culprit in causing gene expression of normal cells to more resemble that of the pathological RS cells by inappropriately activating the JAK/STAT and nuclear factor kappa-light-chain-enhancer of activated B cells (NF-κB) pathways. Activation of these pathways prevented apoptosis in response to stress [40,41]. LMP1 is also able to activate programmed cell death ligand 1 (PD-L1) which inhibits T-cell signaling, allowing for evasion of the immune system [42]. LMP1 also functions to downregulate the shelterin proteins, which in turn can cause telomere dysfunction leading to additional chromosomal mutations [43]. EBV-positive HL can also potentially express LMP2A, which can promote survival of the abnormal BCR negative B cells by mimicking B-cell signaling. Expression of LMP2A can later cause these pretumor cells to transition into RS cells [37,38,44]. A 2012 nested case-control study was looking to see if a specific serological profile had a higher association with developing HL. Further, 128 participants who were diagnosed with HL via histopathological analysis, in situ hybridization, and immunohistochemistry were matched with 368 controls all of whom were chosen from a department of defense repository. Serum from these participants was analyzed in a double-blinded fashion to see how the antibody profile affected risk of malignancy. They concluded that an increase in risk of developing EBV-positive HL was seen when the profile had elevated levels of anti-EBV VCA IgG antibody titer as well as a low ratio of anti-EBNA1 to anti-EBNA2 antibody [45]. While the above study looked for antibodies that could be correlated with development of EBV-positive HL, the next study mentioned looks to see if the presence of the LMP1 protein is predictive of HL development. A 2017 study performed in Pakistan analyzed a total of 66 cases over a period of two years to see how often the LMP1 protein was expressed in EBV-positive HL. They found that 68.1% (with a range of 40-74% among different subtypes of EBV) of their subjects were positive for LMP1 expression concluding that this protein was frequently seen in EBV-positive HL [46]. The results of this study can be contrasted with a 2021 Syrian case-control study that sought to find a less invasive biomarker for EBV-positive HL. They compared 60 cases of HL against 55 matched controls and recorded both LMP1 expression as well as EBV DNA load in the plasma. This study found that only 42% of their cases had a positive LMP1 expression, but plasma EBV load was detectable in all of the EBV-positive cases. Additionally, they saw that higher plasma EBV loads were associated with a worse prognosis [47]. The results of this study can be compared to a cohort study published in 2010 which was also assessing prognostic indicators. This study involved 165 adults, 29 of whom were recorded as having EBV-positive HL via immunohistochemistry staining. Using real-time quantitative PCR analysis, it was then seen that 76% of this group had detectable EBV DNA in their plasma whereas only 2% of the control group produced a similar result, showing that this biomarker may have viability in assessing prognosis. Additionally, this study found that patients receiving therapy for HL showed declining levels of EBV DNA plasma load, which may provide an indicative measure of therapeutic efficacy [48]. Another study published in 2021 also aimed to see if they could find any relationship between EBV status of HL tumors and the overall prognosis of the patient. They analyzed a group of 134 patients who had an untreated HL diagnosis which was tested for EBV positivity by in situ hybridization. This study found no particular association between EBV status and overall survival or failure-free survival, except in groups of over 50 years of age where an EBV-positive status indicated inferior survival statistics [49]. Table 3 includes a summary of the studies which link EBV to HL. 

**Table 3:** Summary of included studies associating Epstein-Barr virus and Hodgkin’s lymphoma. EBV: Epstein-Barr virus; HL: Hodgkin's lymphoma; LMP1: latent membrane protein 1; VCA IgG: immunoglobulin G antibody to viral capsid antigen; EBNA1/EBNA2: Epstein-Barr virus nuclear antigen 1/Epstein-Barr virus nuclear antigen 2

Reference	Design	Population	Cases of Hodgkins	Diagnostic Criteria	Conclusion
Habeeb et al. (2021) [47]	Case-control	115 (60 cases and 55 matched controls)	60	-	Plasma EBV DNA load can be used as a noninvasive biomarker for EBV+ HL
Wang et al. (2021) [49]	-	134 confirmed cases HL	134	In situ hybridization for EBV positivity	Not much association between EBV and survivability factors except in older age groups
Hashmi et al. (2017) [46]	-	66 confirmed cases of HL	66	Histological analysis performed at Liaquat National Hospital	LMP1 is frequently expressed in EBV+ HL. Targeted therapeutic research applications
Levin et al. (2012) [45]	Nested case-control	496 active duty military personnel	128 in the case group (40 were EBV+)	Histological analysis of HL tumors	Increased risk of HL with elevated anti-EBV VCA IgG or low anti EBNA1/EBNA2 antibody
Spacek et al. (2011) [48]	Cohort	165 adults	165 HL, 29 of whom were EBV+	Tissue samples + staining	Plasma EBV DNA load may be valuable for prognosis and patient follow up

Limitations

As with all malignancies, the cancers listed in this study have many risk factors other than just EBV. This study does not address socioeconomic status, dietary habits, environmental hazards, and other factors that could influence EBV and the development of cancer.

## Conclusions

Although EBV is generally associated with infectious mononucleosis, there is a growing amount of research connecting this virus with the development of malignancy, as evidenced by the above studies. Chiefly, the clinical implication of this paper is to further investigate what is known about the viral proteins involved in oncogenesis as well as seek to understand more about EBV’s association with various cancers. This study can serve to concisely explain some of the important aspects of EBV's association with cancer and can serve as an early point of entry for those who are interested in this field. We chose to mention studies that directly measured EBV viral load in people who had commonly associated cancers to highlight just how often EBV positivity is found in certain cancers. This has huge implications given the worldwide prevalence of the virus. The most obvious solution to this problem is for the development of a new vaccine to prevent initial infection and eventual permanent latency. The need for future studies will always be there to monitor to determine prevalence, changes in associations, as well as vaccine efficacy once enough advances in development occur.
